# A polymeric composite protective layer for stable Li metal anodes

**DOI:** 10.1186/s40580-020-00231-w

**Published:** 2020-06-15

**Authors:** Suogang Guo, Li Wang, Yuhong Jin, Nan Piao, Zonghai Chen, Guangyu Tian, Jiangang Li, Chenchen Zhao, Xiangming He

**Affiliations:** 1 College of Chemical Engineering, Beijing University of Chemical Technology, Beijing, 100029 People’s Republic of China; 2 Institute of Nuclear & New Energy Technology, Tsinghua University, Beijing, 100084 People’s Republic of China; 3 Beijing Guyue New Materials Research Institute, Beijing University of Technology, Beijing, 100124 China; 4 Chemical Sciences and Engineering Division, Argonne National Laboratory, Argonne, IL 60439 USA; 5 State Key Laboratory of Automotive safety and Energy, Tsinghua University, Beijing, 100084 People’s Republic of China; 6 School of Chemical Engineering, Beijing Institute of Petrochemical Technology, Beijing, 100176 People’s Republic of China

**Keywords:** AlPO_4_ nanoparticles, fluoride-co-hexafluoropropylene, Protective layer, Lithium metal anode, Secondary lithium batteries

## Abstract

Lithium (Li) metal is a promising anode for high-performance secondary lithium batteries with high energy density due to its highest theoretical specific capacity and lowest electrochemical potential among anode materials. However, the dendritic growth and detrimental reactions with electrolyte during Li plating raise safety concerns and lead to premature failure. Herein, we report that a homogeneous nanocomposite protective layer, prepared by uniformly dispersing AlPO_4_ nanoparticles into the vinylidene fluoride-co-hexafluoropropylene matrix, can effectively prevent dendrite growth and lead to superior cycling performance due to synergistic influence of homogeneous Li plating and electronic insulation of polymeric layer. The results reveal that the protected Li anode is able to sustain repeated Li plating/stripping for > 750 cycles under a high current density of 3 mA cm^−2^ and a renders a practical specific capacity of 2 mAh cm^−2^. Moreover, full-cell Li-ion battery is constructed by using LiFePO_4_ and protected Li as a cathode and anode, respectively, rendering a stable capacity after 400 charge/discharge cycles. The current work presents a promising approach to stabilize Li metal anodes for next-generation Li secondary batteries.

## Introduction

The increasing demand for portable electronics and electric vehicles stimulates the endless pursuit of high-energy–density batteries [1–6]. As promising next-generation batteries, Li/S and Li/O_2_ batteries deliver a high theoretical energy density of 2600 Wh kg^−1^ and 3505 Wh kg^−1^, respectively. It is worth emphasizing that the superior performance of Li/S and Li/O_2_ batteries mainly relies on the merits of metallic lithium (Li) anode. However, the instability of Li/electrolyte interface and undesirable dendritic growth during Li plating/stripping lead to internal short-circuiting and successive thermal runaway, limiting the successful realization of lithium metal batteries (LMBs). Also, the formation of dendrites results in the continuous growth of solid electrolyte interphase (SEI), consuming the liquid electrolyte and leading to premature failure of LMBs. Therefore, different strategies have been developed to inhibit dendrite growth and enhance the lifetime of LMBs [3–5, 7–37].

The uneven surface properties of Li anode, including exposed crystal facets, nonuniform surface composition and roughness, are the root cause of dendrite formation, leading to the uneven transference of Li-ions and nonuniform Li plating/stripping speed. Hence, the local current density is significantly increased, which leads to uneven Li deposition. It has been reported that the localcurrent density can be reduced by using high-surface-area current collectors [38–47], which results in a significantly improved cycling performance. Hence, only a uniform and strong artificial SEI, instead of native SEI, can alter the fundamental self-amplifying behavior of dendritic growth [2, 26, 48–59]. Recently, several research groups aimed to artificially develop a stable SEI to protect Li anode [48–53]. For instance, Huang’s group [60] has prepared an artificial soft-rigid protective layer for dendrite-free Li anodes. It is worth noting Al_2_O_3_ is a popular protective layer for battery applications [61–64]. In particular, the porous Al_2_O_3_ layer, prepared by a facile spin-coating method, acts as a stable and dense interlayer to suppress side reactions between Li metal and electrolyte, and avoids the formation of surface cracks to suppress dendritic growth [62]. However, the inorganic Al_2_O_3_ layers are relatively brittle and the construction method is cumbersome. The elastic moduli of protective layer is important [65]. Therefore, owing to the high elastic moduli of polymeric materials, polymeric SEI layers are being developed to protect Li metal anode and sustain volumetric changes during Li plating and stripping [24, 59, 66, 67]. However, the polymeric layers generally render inferior mechanical strength and ionic conductivity.

On the other hand, the composite artificial films are preferred due to their high ionic conductivity, low interface resistance and high mechanical modulus [3, 68]. Lee et al. [69] have designed an Al_2_O_3_/poly(vinylidene fluoride-co-hexafluoropropylene) (PVDF–HFP) composite layer to protect Li metal anode and demonstrated superior cycling performance in Li/O_2_ batteries. However, Al_2_O_3_ exhibits poor stability in the electrolyte, transforming into AlF_3_ due to the presence of HF and lowering the ionic conductivity. Furthermore, it has been demonstrated that the uniformity of protective layer plays a critical role in inhibiting dendritic growth [3]. Therefore, a chemically and physically stable artificial SEI with high ionic conductivity should be designed to exploit the potential of Li metal anodes.

Nazar et al. have reported that the presence of an ionically conductive and electronically insulative layer on the surface of Li anode significantly suppresses the dendritic growth [70]. Zhang et al. [31, 71] have demonstrated that uniformly distributed Li-ions effectively eliminate the dendrites and lead to dendrite-free Li deposition. The size of Li dendrites is in the range of micrometers [29, 72–74], which implies that the growth of Li dendrites can be suppressed by nanoscale Li plating [75–78]. Previously, we have reported a novel route to uniformly disperse nanoparticles into polymeric solutions [79–83], enhancing the electrochemical performance of LIBs.

Nano-AlPO_4_ composite prepared by organic ligand coordination is reported in our previous publication [84], showing good dispersion in a solution. This inspires us to prepare well-dispersed nano-AlPO_4_ composite film to protect lithium anode. Moreover, a LiF coating layer can be formed via an in situ reaction between Li metal and PVDF-DMF solution to protect lithium metal anode [85].

**Fig. 1:**
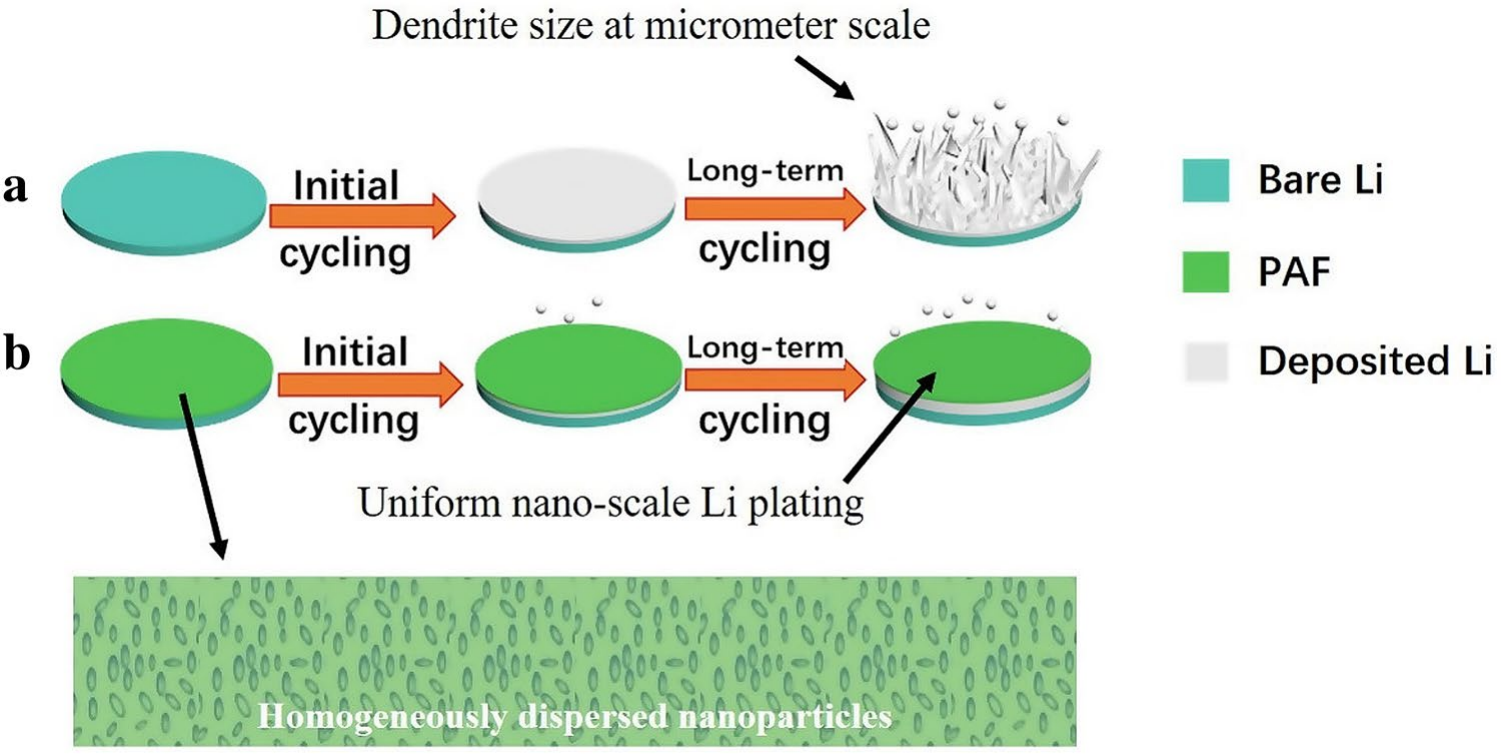
Schematic illustration of Li deposition on **a** bare Li foil during plating, forming micron-sized Li dendrites; and **b** PAF-protected Li foil, where PAF is composed of organic PVDF-HFP and homogeneously dispersive inorganic AlPO_4_ nanoparticles. Consequently, Li-ions are homogeneously redistributed in the film before Li plating, leading to uniform Li plating at nanometer scale

Therefore, we propose a facile process to protect Li anode by forming a well-dispersed nano-AlPO_4_/PVDF-HFP composite film (PAF), rendering good homogeneity and nano-scale uniformity. The results demonstrate that the proposed method effectively suppresses the dendrite growth and leads to superior cycling performance of Li metal anodes. It is worth emphasizing that the usual size of Li dendrites ranges from 1 to 3 μm, as shown in Fig. 1a [29, 72–74]. Owing to homogeneously dispersed nanoparticles in as-proposed PAF, Li-ions are homogeneously redistributed at the nanometer scale and lead to uniform Li-ion plating (Fig. 1b) [32].

## Experimental

### Preparation of PVDF-HFP/AlPO_4_ film

#### Preparation of AlPO_4_ gel-solution

First, Al(NO_3_)_3_·9H_2_O was dissolved in ethanol and mixed with an appropriate amount of P_2_O_5_ in a nitrogen-filled glovebox. The mixed solution was magnetically stirring at 50 °C for 3 h and filtered to collect the supernatant. The supernatant was placed in a water bath at 50 °C and an appropriate amount of (NH_4)2_CO_3_ was completely dissolved by stirring. Then, the mixture was naturally cooled to room temperature and centrifuged at 5000 rpm for 10 min. Finally, the supernatant was separated and diluted with ethanol to obtain the AlPO_4_ concentration of ~ 8 wt%.

#### Fabrication of composite film

First, 0.1 g of PVDF-HFP was dissolved in 5 g of acetone and magnetically stirred for 5 h. Then, 1.25 g of as-prepared AlPO_4_ solution was added into the above mixture and stirred for another 5 h. The obtained mixture was coated on a smooth glass plate with a defined thickness(5 μm). After room-temperature evaporation, the film was peeled-off and cut into circular discs with a diameter of 18 mm. The optimal mass portion of AlPO_4_ in the composite film is 2%.

### Electrochemical characterization

CR2032-type coin cells were assembled in an Ar-filled glovebox with water and oxygen content of < 2 ppm. The coin cells were fabricated by stacking Li foil, PAF-protected Li foil and Celgard2400 separator. The electrochemical characterization was carried out by using Land Battery Tester (CT2001A, Wuhan LAND Electronics Co., Ltd., China). Li|Li and Li|PAF-Li symmetric batteries were cycled at the current densities of 0.5, 2, 3 and 5 mA cm^−2^ in an ether-based electrolyte, i.e., 1.0 mol L^−1^ lithium bis(trifluoromethanesulfonyl) imide (LiTFSI) dissolved in 1,3-dioxolane (DOL)/1,2-dimethoxyethane (DME) (v/v = 1:1) with 1.0 wt% lithium nitrate (LiNO_3_). The capacity of Li plating/stripping was controlled at 1 mAh cm^−2^ or 2 mAh cm^−2^. The full-cell LIBs were constructed by using commercial LiFePO_4_ (LFP) cathode and 1.0 M LiPF_6_-EC/DEC electrolyte. The LFP cathode was prepared by mixing 0.8 g of LFP powder, 0.1 g of Super P conductive carbon black and 1 g of PVDF (10 wt%) binder in NMP solvent. The slurry was coated on Al foil and dried at 60 °C for 2 h, followed by vacuum drying at 120 °C for 12 h. Then, the cathode was pressed by using Rolling Machine (MSK-2150) and punched into circular discs with a diameter of 13.0 mm. The average mass loading of LFP on each disk was ~ 3.4 mg cm^−2^.

### Material characterization

The surface morphology of different Li foils was observed by using a scanning electron microscope (SEM, Hitachi S-4800, Japan). The Young’s modulus of PVDF-HFP/AlPO_4_ composite film was measured by using atomic force microscopy (Bruker Multimode 8 with a Nanoscope V controller) in a N_2_-filled glovebox. X-ray diffraction (XRD) patterns were recorded by using a Rigaku D/MAX 2500/PC X-ray diffractometer, equipped with Cu *Kα* radiations (*λ* = 0.154 nm).

## Results and discussion

Herein, PVDF-HFP is employed due to its excellent film-forming properties and utilization of PVDF-HFP–plasticizer–lithium salt system as a gel electrolyte for LIBs, which can be ascribed to the high Li-ion conductivity at room temperature and good electrochemical stability [86–89]. One should note that PVDF-HFP is a semi-crystalline polymer and the amorphous regions contribute to Li-ion conductivity, whereas crystalline regions ensure mechanical strength [60].

**Fig. 2:**
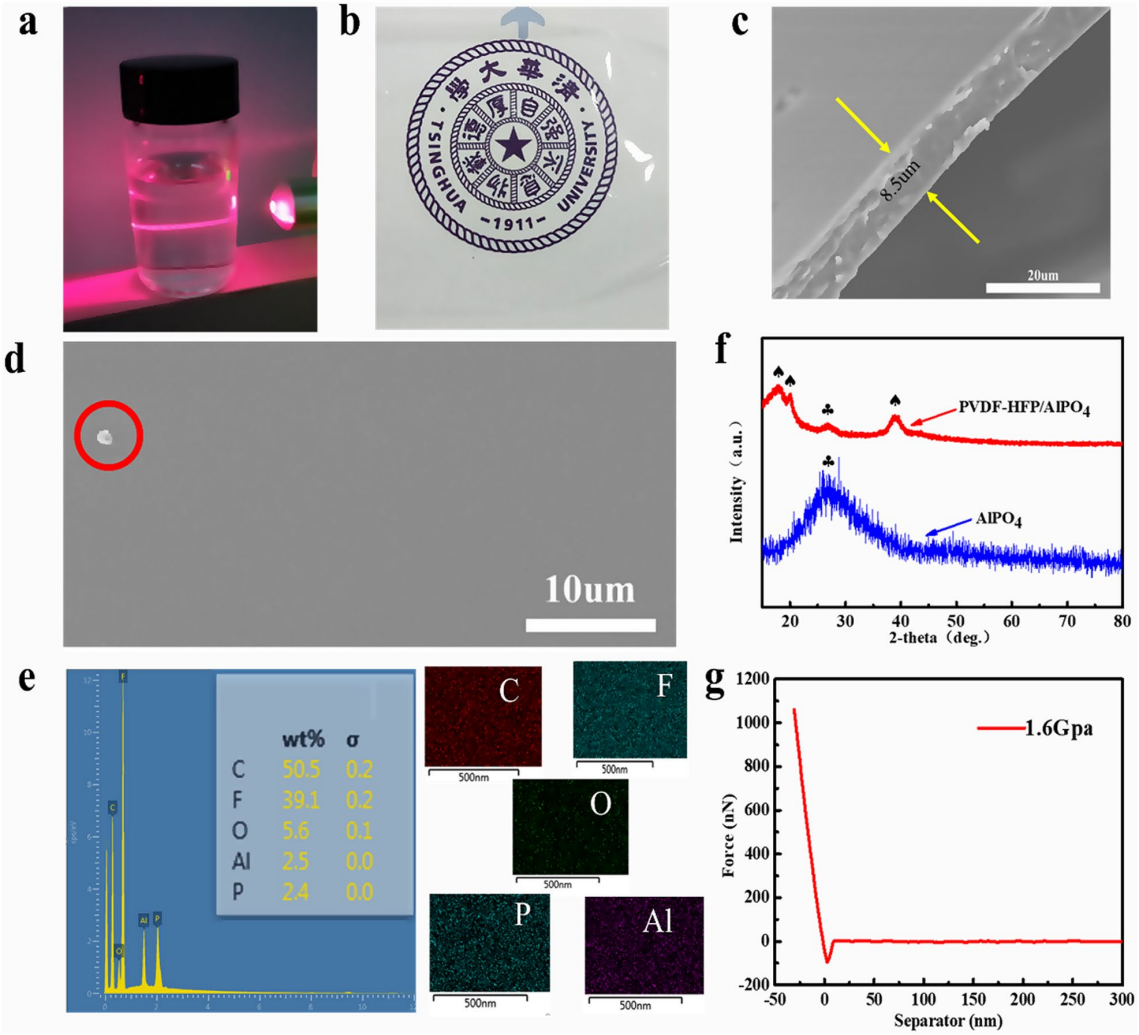
Material characterization: **a** Tyndall effect of PVDF-HFP- AlPO_4_ solution; **b** digital photograph of transparent PAF; **c** cross-sectional and **d** top-view SEM images of PAF; **e** EDS spectrum and element maps of PAF; **f** XRD patterns of pristine AlPO_4_ and PAF; and **g** the force-distance curve and corresponding Young’s modulus of PAF, measured by atomic force microscopy

Figure 2a presents that the as-prepared PVDF-HFP/AlPO_4_ solution exhibits a bright pathway under the action of a laser beam, which is referred as the Tyndall effect and indicates that nanoparticles are well-dispersed in the solution. The as-prepared film is shown in Fig. 2b, exhibiting a transparent appearance and clear texture. It can be concluded that the high content of AlPO_4_ (10 wt%) did not deteriorate the uniformity of PAF, which can be attributed to the homogenous dispersion of nano-sized AlPO_4_ in PVDF-HFP. Furthermore, SEM analysis was carried out to observe the uniformity and flatness of PAF. Figure 2d shows that the film surface is smooth and uniform without any obvious particles or protrusions. The particles, marked by a red-colored circle, correspond to accidentally-deposited dust during film preparation. Moreover, the uniform distribution of AlPO_4_ in PAF can be further confirmed by energy dispersive spectroscopy (EDS). Figure 2e shows uniformly distributed carbon (C) and fluorine (F) elements, originating from PVDF-HFP, whereas oxygen (O), phosphorus (P) and aluminum (Al) represent the presence of AlPO_4_. The weight proportion of O, Al and P was 5.6%, 2.5% and 2.4%, respectively, corresponding to a molar ratio of 4.11:0.95:1.04 (AlPO_4_).

Figure 2f shows XRD patterns of AlPO_4_ powder and PVDF-HFP/AlPO_4_ composite film. The diffraction peaks, located at 2θ = 27.5°, correspond to amorphous phase of AlPO_4_. The diffraction peaks, located at 2θ = 17.9° and 20.0°, correspond to α-phase of PVDF-HFP, whereas the diffraction peak, located at 2θ = 38.9°, represents β-phase PVDF-HFP [90, 91]. XRD results confirm that PAF is a physical mixture of PVDF-HFP and AlPO_4_. In addition, the modulus of inorganic AlPO_4_ is relatively high. Herein, 8.5 μm (Fig. 2c) thick PAF film exhibited Young’s modulus of 1.6 GPa (Fig. 2g), which can be ascribed to the presence of AlPO_4_. On the other hand, Young’s modulus of PVDF-HFP film (12 μm) is only 0.8 GPa [60], which is far lower than the PAF but significantly higher than a typical SEI in LIBs (≈ 150 MPa) [55]. Therefore, the as-prepared PAF, with higher Young’s modulus, is expected to inhibit the growth of Li dendrites during charge/discharge cycling.

**Fig. 3:**
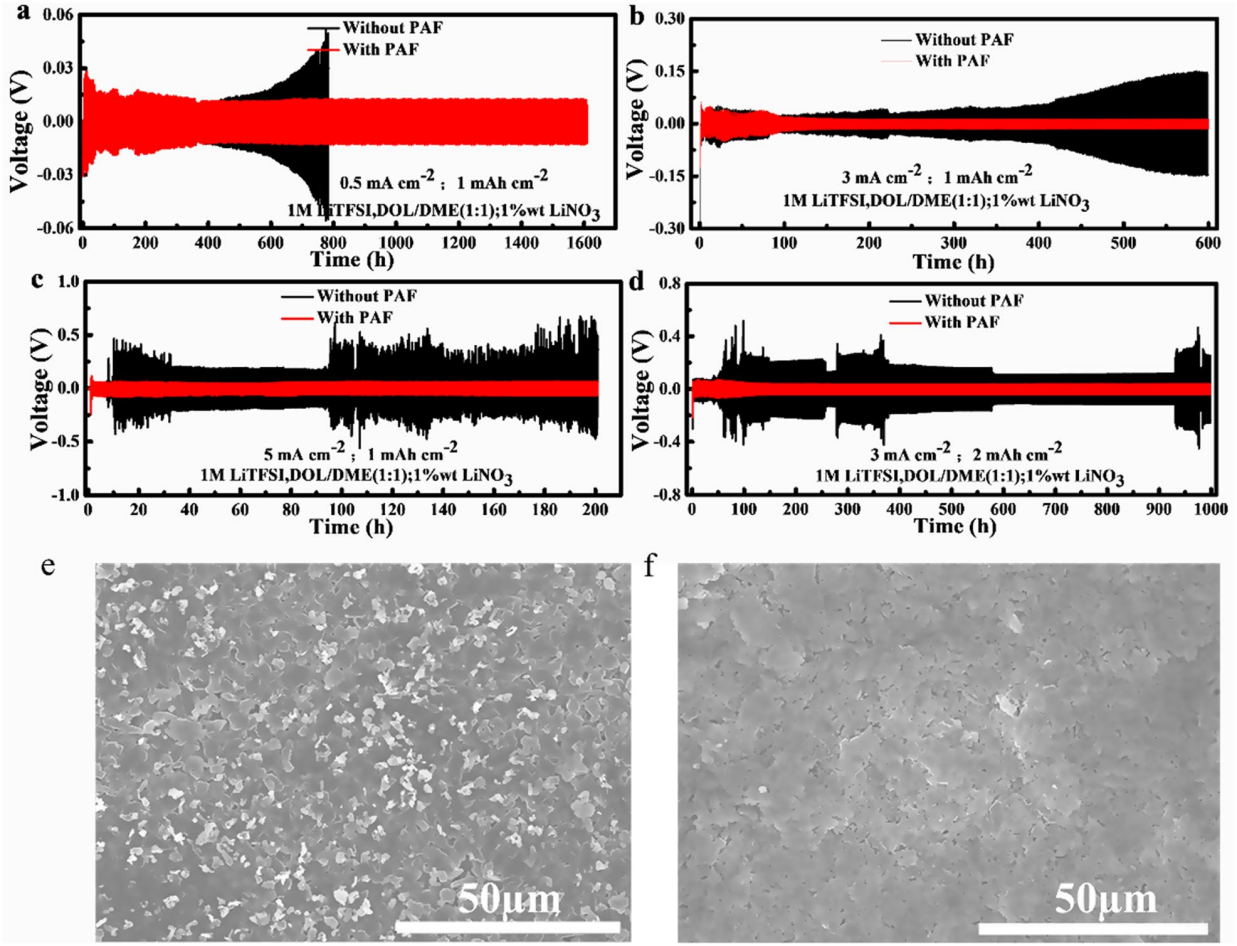
Galvanostatic plating/stripping profiles of symmetric cells at different current densities: **a** 0.5 mA cm^−2^ (capacity: 1 mA cm^−2^); **b** 3 mA cm^−2^ (capacity:1 mA cm^−2^); **c** 5 mA cm^−2^ (capacity:1 mA cm^−2^) and **d** 3 mA cm^−2^ (capacity: 2 mA cm^−2^). SEM images of **e** bare Li foil and **f** PAF-protected Li foil after 200 cycles at 5 mA cm^−2^ (capacity: 1 mA cm^−2^)

Briefly, PAF is expected to work as a passivation layer and minimize the side reactions between metallic Li and liquid electrolyte. Consequently, symmetrical cells, with PAF-protected Li anodes, outperformed the symmetrical cells with bare Li at different current densities (Fig. 3a–d). The PAF-protected Li anode exhibited long-term cycling of up to 1600 h at a low current density of 0.5 mA cm^−2^. As shown in Fig. 3a, PAF-protected Li anode exhibits higher electrochemical stability with lower voltage hysteresis than the bare Li electrode. The overpotential of the bare Li anode started to gradually increase after 125 cycles (500 h), whereas the overpotential of PAF-protected Li anode remained stable even after 4000 cycles (1000 h). After 1600 h of charge/discharge time, the overpotential (281 mV) of the bare Li anode is found to be 3 times higher than the PAF-protected Li anode (87 mV). When the current density was increased to 3 mA cm^−2^, the overpotential of bare Li anode started to gradually increase after 100 h and exhibited an overpotential of 147.9 mV after 600 h.

Such a huge hysteresis indicates the formation of a highly resistive interfacial layer, consisting of dead Li and the consumed electrolyte, which lead to continuous breaking and reconstruction of the SEI layer. On the other hand, the overpotential of PAF-protected Li anodes remained lower than 13 mV during the initial 600 h of operation, which is consistent with the low current density. The lower overpotential and stable performance can be attributed to the protection of Li anode by PAF.

Interestingly, the phenomenon became more prominent at higher current densities, i.e., 5 mA cm^−2^ (Fig. 3c). Under such a high current rate of 5 mA cm^−2^, the cell voltage of bare Li anode rapidly increased after 25 cycles and remained unstable during subsequent cycles, whereas PAF-protected Li anode still exhibited a low polarization voltage (61 mV) after 500 cycles (200 h). Moreover, the cell with PAF-protected Li anode delivered a high areal capacity of 2 mAh cm^−2^ at the current density of 3 mA cm^−2^, which shows better performance than the bare Li anode. Figure 3e, f present SEM images of bare and PAF-protected Li anodes, respectively, after 200 cycles at a current density of 5 mA cm^−2^ and a plating/striping capacity of 1 mAh cm^−2^. The SEM image of bare Li anode shows several particles on the surface of Li foil after cycling, indicating the deposition of a large amount of dead lithium and some Li dendrites. However, the PAF-protected Li anode did not show the dead Li or dendrites after electrochemical cycling (Fig. 3f).

**Fig. 4:**
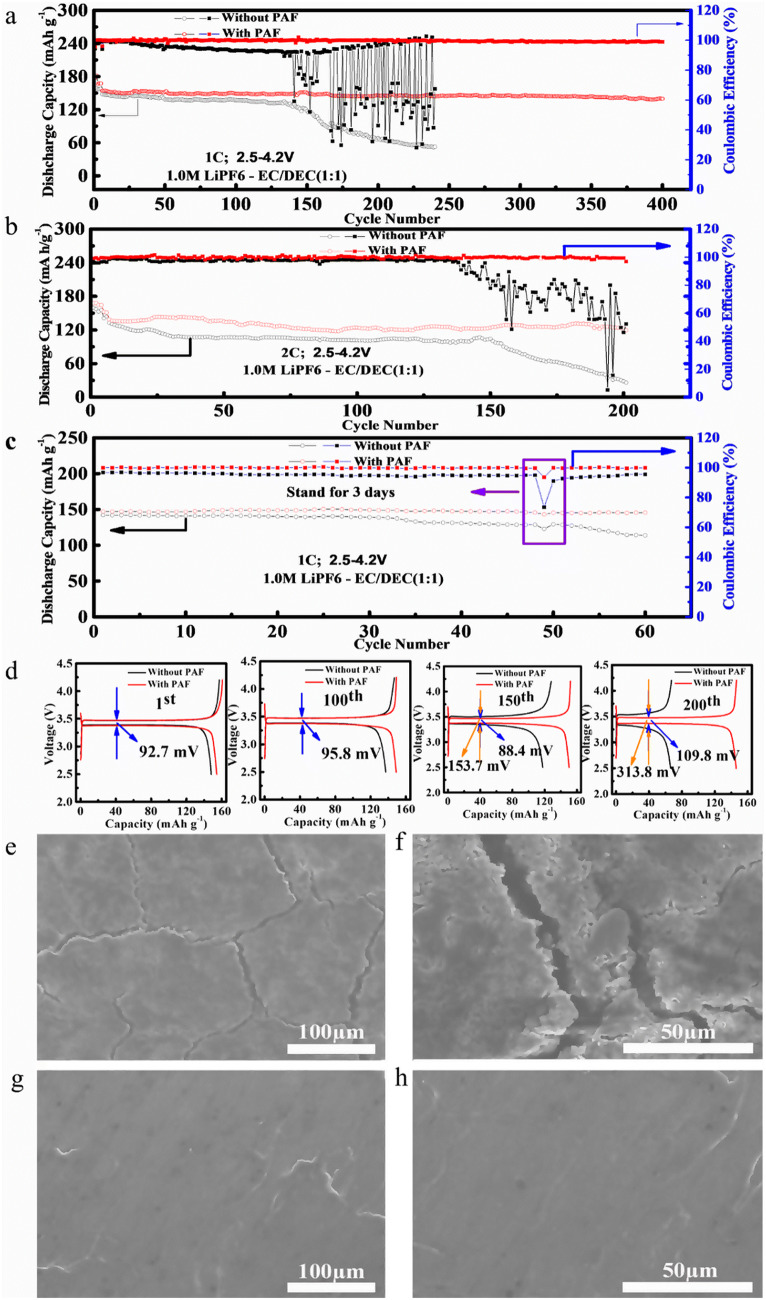
Electrochemical performance of full-cell LIBs and SEM images of cycled Li metal anodes: long-term cycling stability at **a**, **c** 1 C and **b** 2 C; **d** galvanostatic charge–discharge profiles at 1st, 100th, 150th and 200th cycle; SEM images of **e**, **f** bare Li and **g**, **h** PAF-protected Li foils after 200 charge/discharge cycles

Furthermore, we have assembled full-cell LIBs by using LFP, as a cathode, and PAF-protected Li metal, as an anode, to demonstrate the potential of using the proposed coating methodology in practical applications. Figure 4 shows the cycling performance of full-cell LIB and morphology of Li anode after charge/discharge cycling. Figure 4a, b present the cycling performance of Li|LFP and PAF-Li|LFP full-cells, respectively, at the 1 C and 2 C (1 C = 140 mAh g^−1^). The Li|LFP full-cell LIB delivered an initial specific capacity of 148.1 mAh g^−1^ at 1 C, however, the capacity drastically decreased to 118 mAh g^−1^ after 150 charge/discharge cycles, corresponding to a capacity retention of 79.7%. One should note that Li|LFP full-cell LIB exhibited a capacity of 52.2 mAh g^−1^ after 240 charge/discharge cycles. However, PAF-Li|LFP full-cell LIB rendered an initial specific capacity of 154.3 mAh g^−1^ at 1 C, which is slightly higher than the Li|LFP full-cell LIB, and retained 90% of the initial capacity after 400 charge/discharge cycles (140 mAh g^−1^).

Furthermore, we have carried out an interesting experiment, where the full-cell LIBs were galvanostatically cycled at 1 C and shelf-stored for 3 days after the 49th cycle. Interestingly, both full-cell LIBs exhibited a capacity drop. The drop in discharge capacity of Li|LFP full-cell LIB is significantly lower than the drop in discharge capacity of PAF-Li|LFP full-cell LIB. Subsequently, the capacity of Li|LFP full-cell LIB started to rapidly decrease after the 49th cycle, whereas the capacity of PAF-Li|LFP full-cell LIB is restored to the pre-49th cycle value (Fig. 4c). This shows the promise of PAF in maintaining the cycling stability of full-cell LIBs.

Moreover, the voltage curves show that both full-cell LIBs exhibit the same polarization voltage (92.7 mV) during the initial cycle (Fig. 4d). Similarly, after 100 charge/discharge cycles, the polarization voltage of both full-cell LIBs exhibits similar values. However, Li|LFP full-cell LIB shows a large polarization voltage of 153.7 mV and 313.8 mV after 150 and 200 cycles, respectively, whereas the PAF-Li|LFP full-cell LIB still exhibits a low polarization voltage of 109.8 mV after 200 cycles, corresponding to low interfacial resistance of PAF-protected Li anode. The surface morphology (Fig. 4 e–h) of electrochemically-cycled Li anode, after 230 charge/discharge cycles, shows that the rough surface of bare Li foil possesses a large number of cracks (Fig. 4e, f), whereas PAF-protected Li foil exhibits a smooth and dense surface (Fig. 4g, h). One should note that the presence of PAF protective layer avoids the consumption of metallic Li due to side reactions between Li and electrolyte during long-term cycling.

**Fig. 5:**
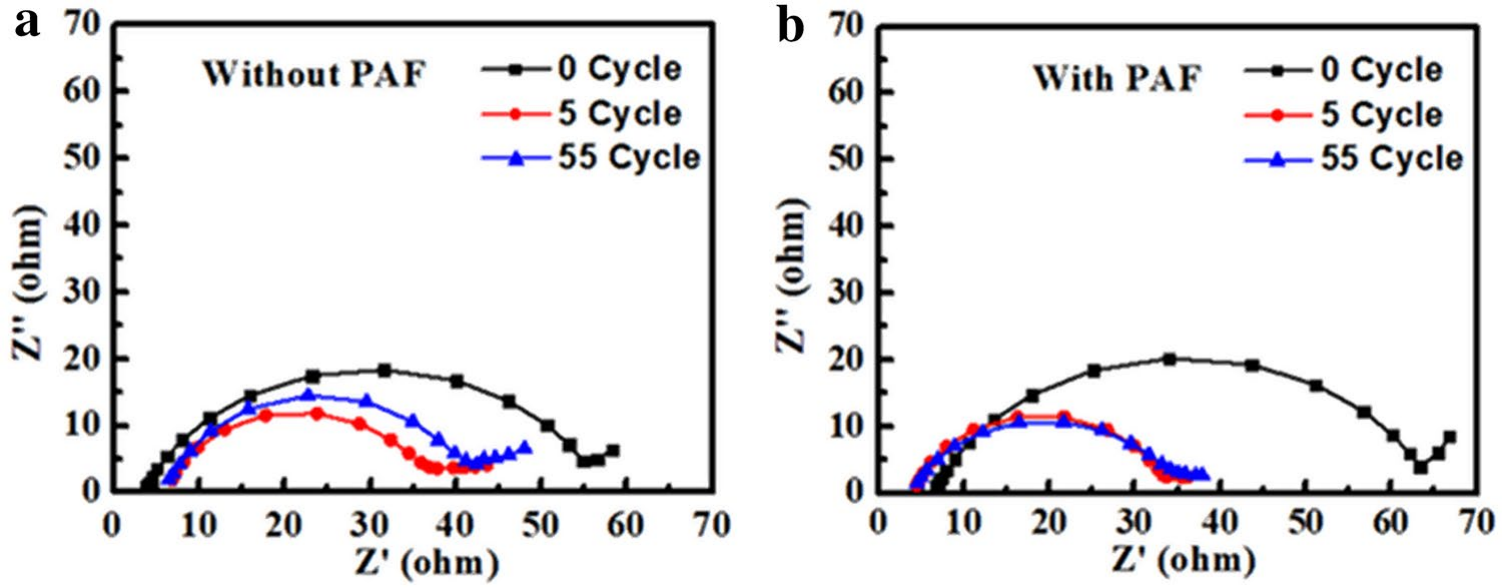
Electrochemical impedance spectra of **a** Li/LFP full-cell LIB and **b** PAF-Li/LFP full-cell LIB after charge/discharge cycling at 1 C

Therefore, we have also carried out electrochemical impedance spectroscopy to understand the charge transfer kinetics of Li and PAF-protected Li anodes, as shown in Fig. 5. It should be noted that the high-frequency semicircle represents the interfacial resistance at the electrode/electrolyte interface, which also reflects the stability of SEI layer. Prior to electrochemical cycling, the PAF-Li|LFP full-cell LIB exhibits a higher interfacial resistance of 64 Ω, whereas Li|LFP full-cell LIB exhibits an interfacial resistance of only 55 Ω, which can be attributed to the presence of PAF. However, the impedance of PAF-Li|LFP full-cell LIB has dropped to 35 Ω after 5 cycles (Fig. 5b). In subsequent cycles, the impedance of Li|LFP full-cell LIB increased, whereas the impedance of PAF-Li|LFP full-cell LIB remained similar. One should note that the increase in impedance can be attributed to the parasite reactions between Li metal and electrolyte, resulting in SEI growth. The stable impedance of PAF-protected Li anode indicates that the presence of an artificial SEI avoids the abovementioned problems.

## Conclusions

In summary, we have demonstrated that a composite membrane, composed of inorganic and organic components, effectively protects the Li metal anode during electrochemical cycling by hindering the dendritic growth and suppressing the side reactions. The PAF composite film, with PVDF-HFP to AlPO_4_ mass ratio of 9:1 and a thickness of 8.5 μm, exhibited Young’s modulus of 1.6 GPa, which is far higher than a typical SEI (≈ 150 MPa).

Moreover, full-cell Li-ion battery was constructed by using LiFePO_4_ cathode and PAF-protected Li anode, which rendered a high specific capacity of 148.1 mAh g^−1^ and excellent capacity retention of 90% after 400 charge/discharge cycles. The results reveal that PAF-protected Li anodes exhibited a low over-potential of 375 mV and stable cycling performance (> 1000 h) under a high current density of 3 mA cm^−2^ and high areal capacity of 2 mAh cm^−2^. Thus, we anticipate that the design of an organic–inorganic hybrid artificial protective film is an industrially viable option for the development of next-generation secondary Li batteries.

## Data Availability

The test materials and data are available from the corresponding author on reasonable request.

## Abbreviations

Al: Aluminum
C: Carbon
DOL: 1,3-dioxolane
DME: 1,2-dimethoxyethane
EDS: Energy dispersive spectroscopy
F: Fluorine
Li: Lithium
LiTFSI: Lithium bis(trifluoromethanesulfonyl) imide
LFP: LiFePO_4_
LMBs: Lithium metal batteries
O: Oxygen
P: Phosphorus
PVDF − HFP: Poly(vinylidene fluoride-co-hexafluoropropylene)
PAF: PVDF-HFP/nano-AlPO_4_ composite film
SEI: Solid electrolyte interphase
XRD: X-ray diffraction
